# Functional roles of xylanase enhancing intestinal health and growth performance of nursery pigs by reducing the digesta viscosity and modulating the mucosa-associated microbiota in the jejunum

**DOI:** 10.1093/jas/skac116

**Published:** 2022-04-11

**Authors:** Vitor Hugo C Moita, Marcos Elias Duarte, Sung Woo Kim

**Affiliations:** Department of Animal Science, North Carolina State University, Raleigh, NC 27695, USA

**Keywords:** apparent ileal digestibility, intestinal health, nursery pigs, oxidative stress, viscosity, xylanase

## Abstract

This study was conducted to investigate the functional roles of an endo-β-1,4-xylanase on the intestinal health and growth performance of nursery pigs. A total of 60 pigs (21 d old, 6.9 ± 0.8 kg body weight [**BW**]) were allotted based on a randomized complete block design with sex and initial BW as blocks. Dietary treatments had nutrients meeting the requirements with increasing levels of endo-β-1,4-xylanase (0, 220, 440, 880, 1,760 xylanase unit [**XU**] per kg feed) and fed to pigs in three phases (phases 1, 2, and 3 for 10, 14, and 14 d, respectively). Titanium dioxide (0.4%) was added to the phase 3 diets as an indigestible marker. On day 38, all pigs were euthanized to collect ileal digesta to measure apparent ileal digestibility (**AID**), jejunal digesta to measure viscosity, and jejunal mucosa to evaluate intestinal health. Data were analyzed using the MIXED procedure for polynomial contrasts and the NLMIXED procedure for broken line analysis of SAS. Increasing xylanase in the nursery diets reduced (linear, *P* < 0.05) the digesta viscosity in the jejunum. Increasing xylanase tended to reduce the relative abundance of *Cupriavidus* (*P* = 0.073) and *Megasphaera* (*P* = 0.063); tended to increase the relative abundance of *Succinivibrio* (*P* = 0.076) and *Pseudomonas* (*P* = 0.060); and had a quadratic effect (*P* < 0.05) on the relative abundance of *Acinetobacter* (maximum: 2.01% at 867 XU per kg feed). Xylanase from 0 to 1,087 XU per kg feed reduced (*P* < 0.05) jejunal malondialdehyde. Xylanase from 0 to 1,475 XU per kg feed increased (*P* < 0.05) the AID of neutral detergent fiber. Increasing xylanase increased (*P* < 0.05) the AID of ether extract and tended to increase (*P* = 0.058) the AID of crude protein. Increasing xylanase did not affect growth performance on overall period, whereas xylanase from 0 to 736 XU per kg feed increased (*P* < 0.05) average daily gain (**ADG**) during days 31 to 38. In conclusion, xylanase supplementation showed benefits on intestinal health by reducing digesta viscosity, the relative abundance of potentially harmful bacteria, and the oxidative stress in the jejunal mucosa, collectively enhancing intestinal morphology and the AID of nutrients. Xylanase supplementation at a range of 750 to 1,500 XU per kg feed provided benefits associated with reduced oxidative stress, increased nutrient digestibility, resulting in potential improvement on growth performance of nursery pigs by increasing the average daily feed intake and moderately improving the ADG throughout the last week of feeding.

## Lay Summary

Cereal grains and by-products from cereal processing are extensively used in diets for pigs. These feedstuffs contain soluble fiber that makes digesta viscous in the small intestine. Increased digesta viscosity interferes with the digestion process, changes the ecosystem of bacteria on the mucosal lining of the small intestine, and impairs the intestinal health of young pigs. Supplemental enzymes targeting soluble fiber have been used in feeding young pigs in order to remove the negative impacts of soluble fiber on nutrient utilization and intestinal health. This study used the enzyme xylanase that specifically targets xylan and arabinoxylan largely present in corn and corn by-products. The aim of this study was to investigate how effectively this xylanase work in the small intestine of young pigs by reducing digesta viscosity, positively modulating the bacterial ecosystem on the mucosal lining of the small intestine, improving intestinal health, nutrient digestibility, and finally supporting growth. Xylanase supplementation to feeds for nursery pigs showed benefits on intestinal health by reducing digesta viscosity, oxidative stress, and potentially harmful bacteria in the jejunal mucosa, collectively enhancing intestinal morphology and nutrient digestibility.

## Teaser

Dietary supplementation of xylanase at a range of 750 to 1,500 xylanase unit per kg feed reduced the abundance of potentially harmful bacteria in the jejunal mucosa, oxidative damages in the jejunum, and ileal digestibility of nutrients of nursery pigs.

## Introduction

There are different challenges relative to the early weaning process that can impact the subsequent performance of pigs, such as nutritional, environmental, and physiological adaptions (Campbell et al., 2013; Moeser et al., 2019). Upon weaning, pigs start to consume feed including plant-based feedstuffs that contain antinutritional factors, such as allergenic proteins, nonstarch polysaccharides (**NSP**), flatulence-producing compounds, and phytate (Kim et al., 2003; Taliercio and Kim, 2013; Humer et al., 2015).

The cereal grains and by-products used in pig diets can be classified into viscous and nonviscous regarding the amounts and structure of soluble NSP in their composition and their impacts on the physical-chemical properties of digesta (Choct, 2006, 2015). The most common soluble NSP found in corn–soy-based pig diets are xylan and arabinoxylan, present in corn and distillers’ dried grains with soluble (**DDGS**; Choct, 2015; Baker et al., 2021), and xyloglucan present in soybean meal (Irish and Balnave, 1993; Bach Knudsen, 2014). The amount of NSP in DDGS accounts for approximately 33%, with xylans being the primary NSP component (Pedersen et al., 2014). Moreover, during ethanol production, the structure of xylans in corn is affected in multiple manufacturing steps increasing the solubility resulting in increased negative impacts on digesta viscosity and nutrient digestibility (Pedersen et al., 2014).

High amounts of soluble NSP can contribute to the increase of digesta viscosity and bulkiness due to its water holding capacity that also affects the passage rate (Tervilä-Wilo et al., 1996; O’Neill et al., 2012; Passos et al., 2015; Chen et al., 2020). On the other hand, the insoluble NSP has been described as affecting intestinal mobility and digesta transit time by acting as a barrier for other endogenous digestive enzymes, such as amylases and proteases (Choct, 2006, 2015).

Xylanase is classified as a carbohydrase enzyme that is capable of hydrolyzing the xylan structure in feedstuffs. Important benefits have been reported in the improvement of growth performance, intestinal health, and nutrient digestibility related to the supplementation of xylanase in pig diets (Passos et al., 2015; Tiwari et al., 2018; Duarte et al., 2019; Chen et al., 2020). The supplementation of xylanase contributes to the depolymerization of the xylan structure into shorter chains and the breaking down of the cell wall matrix (Choct, 2015; Petry and Patience, 2020; Baker et al., 2021). As a result, it favors the reduction of digesta viscosity and aids in the release of entrapped nutrients by facilitating the access of digestive enzymes to their substrates within the short feed transit time to improve nutrient digestibility (de Lange et al., 2010; Kiarie et al., 2013; Tiwari et al., 2018).

The use of xylanase in pig diets may provide benefits that go beyond the reduction of antinutritional factors associated with the presence of xylans and improving nutrient digestibility and growth performance. It may also play functional roles on the intestinal health by reducing the digesta viscosity that in turn will generate forms of more fermentable NSP-released compounds, such as xylooligosaccharides, that can positively impact the relative abundance and diversity of the intestinal microbiota (de Lange et al., 2010; Jha and Berrocoso, 2015; Duarte et al., 2020; Baker et al., 2021). A positive modulation of the intestinal microbiota is directly associated with the intestinal health, well-being, and subsequent performance of the pigs throughout their productive life (Duarte and Kim, 2021).

It was therefore, hypothesized that supplemental xylanase could play functional roles on the intestinal health and growth performance of nursery pigs by decreasing the digesta viscosity in the small intestine and modulating the mucosa-associated microbiota in the jejunum. This study was conducted to evaluate the functional roles of endo-β-1,4-xylanase on the intestinal health and growth performance of nursery pigs possibly by reducing the digesta viscosity in the small intestine and positively modulating the mucosa-associated microbiota in the jejunum.

## Materials and Methods

The experimental protocol was approved by the Institutional Animal Care and Use Committee of North Carolina State University.

### Animal, design, and diets

The experiment was conducted at the Metabolism Educational Unit at North Carolina State University (Raleigh, NC). A total of 60 newly weaned pigs at 21 d of age (30 barrows and 30 gilts) with an initial body weight (**BW)** of 6.9 ± 0.8 kg were allotted to 5 dietary treatments based on a randomized complete block design with initial BW and sex as blocks. The dietary treatments were a basal diet formulated meeting the nutrient requirements of NRC (2012) and the basal diet supplemented with increasing levels (0, 220, 440, 880, and 1,760 xylanase unit [**XU**] per kg feed) of an endo-β-1,4-xylanase (GH10; CJ BIO, Seoul, South Korea). The genetic information of xylanase originated from *Orpinomysis* PC2 and the enzyme was produced by *Trichoderma reesei*. It has no other declared enzyme activities in the composition and is insensitive to *Triticum aestivum* xylanase inhibitor and xylanase inhibitor protein. Pure xylanase was premixed with corn and added to the basal diets as shown in Table 1. The calculated and analyzed enzyme activities in the tested mixtures are shown in Table 2. One unit of xylanase activity is defined as the amount of enzyme required to release 1 µM of 4-nitrophenol from the XylX6 substrate in 1 min at 40 °C in 100 mM sodium phosphate buffer, pH 6.0, and is termed a XU.

**Table 1.** Composition of experimental diets^1^ (as-fed basis)

Item	Phase 1	Phase 2	Phase 3
Feedstuff, %			
Corn, yellow dent	40.05	38.43	40.52
Soybean meal, 48% CP	19.00	21.50	24.00
Corn, DDGS^2^	0.00	15.00	30.00
Whey permeate	20.00	12.00	0.00
Blood plasma	4.00	1.60	0.00
Fish meal	4.00	2.00	0.00
Poultry meal	10.00	5.00	0.00
Poultry fat	1.30	2.00	1.80
l-Lys HCl	0.31	0.40	0.45
dl-Met	0.18	0.11	0.04
l-Thr	0.06	0.05	0.05
Salt	0.23	0.22	0.22
Vitamin premix^3^	0.03	0.03	0.03
Trace mineral premix^4^	0.15	0.15	0.15
Dicalcium phosphate	0.00	0.20	0.62
Limestone	0.19	0.80	1.21
Titanium dioxide	0.00	0.00	0.40
Xylanase premix^5^	0.50	0.50	0.50
Calculated composition			
DM, %	90.81	90.30	89.21
ME, kcal/kg	3,436	3,428	3,376
CP, %	25.23	23.99	23.50
EE, %	5.26	6.73	7.47
SID^6^ Lys, %	1.50	1.35	1.23
SID Cys + Met, %	0.82	0.74	0.68
SID Trp, %	0.26	0.23	0.21
SID Thr, %	0.88	0.79	0.73
AX^7^, %	2.69 (0.41)	5.46 (1.18)	8.36 (1.96)
Ca, %	0.85	0.80	0.70
STTD^8^ P, %	0.49	0.40	0.33
Total P, %	0.74	0.64	0.57
Analyzed composition, % (as DM basis)			
DM	89.29	88.47	87.35
CP	24.34	23.23	23.14
EE	5.02	5.24	5.39
ADF	2.93	3.70	5.25
NDF	8.20	9.91	13.09
Ca	0.97	0.87	0.79
Total P	0.82	0.69	0.65

Diets in each phase were supplemented with increasing supplementation of 0, 30, 60, 120, and 240 g/ton of xylanase or 0, 220, 440, 880, and 1,760 XU per kg feed.

DDGS, distillers dried grains with solubles.

The vitamin premix provided per kg of complete diet: 6,614 IU of vitamin A as vitamin A acetate, 992 IU of vitamin D_3_, 19.8 IU of vitamin E, 2.64 mg of vitamin K as menadione sodium bisulfite, 0.03 mg of vitamin B_12_, 4.63 mg of riboflavin, 18.52 mg of d-pantothenic acid as calcium panthonate, 24.96 mg of niacin, and 0.07 mg of biotin.

The trace mineral premix provided per kg of complete diet: 33 mg of Mn as manganous oxide, 110 mg of Fe as ferrous sulfate, 110 mg of Zn as zinc sulfate, 16.5 mg of Cu as copper sulfate, 0.30 mg of I as ethylenediamine dihydroiodide, and 0.30 mg of Se as sodium selenite.

Xylanase enzyme mixed with corn.

SID, standardized ileal digestibility.

AX, arabinoxylan values were given by Bach Knudsen (2014) and Tiwari et al. (2018). Values inside parentheses denote the soluble portion of NSP. Values outside parentheses denote the insoluble portion of NSP.

STTD, standardized total tract digestible.

**Table 2.** Xylanase activity in the feed (mean ± SE)

Item	Xylanase, g/ton				
	0	30	60	120	240
Calculated activity, XU per kg of feed^1^	0	220	440	880	1,760
Analyzed activity, XU per kg of feed					
Phase 1	17 ± 8	297 ± 97	391 ± 6	748 ± 56	1,675 ± 281
Phase 2	74 ± 24	163 ± 18	413 ± 73	726 ± 93	1,680 ± 288
Phase 3	4 ± 1	202 ± 65	401 ± 19	753 ± 66	1,636 ± 133

One unit of enzyme activity is defined as the amount of enzyme required to release 1 µM of 4-nitrophenol from the XylX6 substrate in 1 min at 40 °C in 100 mM sodium phosphate buffer, pH 6.0, and is termed a XylX6 Unit (XU). The analyzed enzyme activity in the product was 7,437 ± 351 XU per g.

Pigs were housed individually in a pen and had free access to feeds and water. Individual housing was essential to quantify the impact of the main effect on response parameters (Wishart, 1939; Shen et al., 2012; Passos et al., 2015). The experimental period was 38 d, which was divided into three dietary phases: phase 1 (days 1 to 10), phase 2 (days 11 to 24), and phase 3 (days 25 to 38). The dietary phases were established according to the BW of the pigs. The BW and feed intake were recorded at the end of each week to calculate the average BW, average daily gain (**ADG**), average daily feed intake (**ADFI**), and G:F as indicators of growth performance. In phase 3, titanium dioxide (0.4%) was added to the diets as an indigestible external marker to further determine the apparent ileal digestibility (**AID**) of nutrients.

### Sample collection and processing

After 38 d of feeding, all the pigs were euthanized to remove the gastrointestinal tract to collect digesta from mid-jejunum (3 m after the pyloric duodenal junction; Cheng et al., 2021) to measure viscosity; mucosa from mid-jejunum to characterize microbiota composition, inflammatory, and oxidative stress parameters; mid-jejunal tissues to measure morphology and crypt cell proliferation; and ileal digesta (a portion of 30 cm prior to the ileocecal valve) to measure the AID of nutrients. Digesta from mid-jejunum was collected into falcon tubes (50 mL), placed on ice, and immediately carried to the lab to measure viscosity. Mucosal samples from mid-jejunum were scraped, placed into 2 mL tubes, and later stored at −80 °C (after snap-freezing in liquid nitrogen, immediately after collection) for the microbiome, inflammatory, and oxidative stress analysis. Sections (5 cm) of the mid-jejunum were taken, flushed with a 0.9% saline solution, and placed into 50 mL tubes with 40 mL of 10% neutral buffered formalin to be fixed for further microscopic assessment of jejunal morphology. For measuring the AID of dry matter (**DM**), crude protein (**CP**), ether extract (**EE**), neutral detergent fiber (**NDF**), acid detergent fiber (**ADF**), and gross energy (**GE**), the ileal digesta was collected into 150 mL containers and placed on ice, and then stored at −20 °C for further analysis. The sample collection procedures were performed as previously described (Duarte et al., 2019).

About 1 g of frozen jejunal mucosa was taken with 2 mL phosphate-buffered saline solution (**PBS**) into 5 mL polypropylene tubes. Mucosa samples were ground 30 s on ice using a tissue homogenizer (ThermoFisher Scientific, Waltham, MA) and transferred to new 2 mL microcentrifuge tubes for centrifugation for 15 min at 14,000 × *g*. The supernatant was collected into 8 sets of 0.5 mL polypropylene tubes and stored at −80 °C for further analysis. The sample preparation procedures for analysis were performed as previously described (Cheng et al., 2021).

### Digesta viscosity

Following the procedure by Passos et al. (2015) and Duarte et al. (2019), digesta collected from the mid-jejunum was divided into two tubes (15 mL) and centrifuged at 1,000 × *g* for 10 min at 4 °C to obtain the liquid phase. The supernatant of each tube was collected into 2 mL tubes and centrifuged at 10,000 × *g* at 4 °C for 10 min. The supernatant obtained was transferred to another 2 mL tube and kept on ice for further assessment of viscosity. The amount of 0.5 mL of digesta supernatant was placed in the viscometer (Brookfield Digital Viscometer, Model DV-II Version 2.0, Brookfield Engineering Laboratories Inc., Stoughton, MA) set at 25 °C. The viscosity measurement was the average between 45.0 and 22.5 per s shear rates, and the viscosity values were recorded as apparent viscosity in millipascal-seconds (mPa.s).

### Relative abundance and diversity of the mucosa-associated microbiota in jejunum

The DNA was extracted from jejunal mucosa samples for microbiome analysis, as previously described by Duarte et al. (2020). The QIAamp Fast DNA Stool Mini kit (#51604, Qiagen; Germantown, MD) was used to perform the DNA extraction. Samples of extracted DNA were sent to Mako Medical Laboratories (Raleigh, NC) for microbial sequencing using the 16S rRNA technique. The samples were prepared for template using the Ion Chef instrument and sequencing was performed on the Ion S5 system (ThermoFisher Scientific). The Ion 16S Metagenomics Kit 113 (ThermoFisher Scientific) was used to amplify variable regions V2, V3, V4, V6, V7, V8, and V9 of the 16S rRNA gene. To produce raw unaligned sequence data files of the relative abundance, sequences were processed using the Torrent Suite Software (version 5.2.2; ThermoFisher Scientific). The Ion Reporter Software Suite (version 5.2.2) of bioinformatics analysis tools (ThermoFisher Scientific) was used to perform the sequence data analysis, alignment to GreenGenes and MicroSeq databases, alpha and beta diversity plot generation, and the operational taxonomic unit (**OTU**) table generation. The Ion Reporter’s Metagenomics 16S workflow powered by QIIME (version w1.1) was used to analyze the samples. The relative abundance for phylum, family, species, and genus was calculated based on the OTU data as previously described (Kim et al., 2019). The “Others” were considered representing the combined OTU with a relative abundance <1%.

### Inflammatory and oxidative stress parameters

The concentrations of total protein, tumor necrosis factor-alpha (TNF-α), interleukin 8 (IL-8), malondialdehyde (**MDA**), and protein carbonyl (**PC**) were measured by the colorimetric method using commercially available kits according to instructions of the manufacturers. The absorbance was read using an ELISA plate reader (Synergy HT, BioTek Instruments, Winooski, VT) and software (Gen5 Data Analysis Software, BioTek Instruments). Mucosa samples were diluted (1:40) in working range of 0 to 2,000 μg/mL for the measurement of total protein using Pierce BCA Protein Assay Kit (#23225, ThermoFisher Scientific). The amount of 25 μg/mL of each sample and standards was pipetted into a microplate well. The BCA working reagent (200 μg/mL) was added to each well and incubated at 37 °C for 30 min. Then, the absorbance was measured at 562 nm. The concentration was calculated based on the standard curve created from the concentration and absorbance of the respective standard and further used to normalize the concentration of other parameters.

The TNF-α was measured following Porcine TNF-α Immunoassay Kit (#PTA00, R&D Systems; Minneapolis, MN). The working range of standards was 0 to 1,500 pg/mL and the absorbance were read at 450 and 550 nm. The concentrations of TNF-α were calculated based on the standard curve created from the concentration and absorbance of the respective standard and described as pg/mg protein, as previously described (Chaytor et al., 2011). The IL-8 was measured following Porcine IL-8/CXCL8 Immunoassay Kit (#P8000, R&D Systems). For this analysis, mucosa samples were diluted (1:20) in a working range of 0 to 4,000 pg/mL and the absorbance were read at 450 and 550 nm. The concentrations of IL-8 were calculated based on the standard curve created from the concentration and absorbance of the respective standard and described as ng/mg protein, as previously described (Duarte et al., 2020).

MDA was measured following OxiSelect TBARS MDA Quantitation Assay Kit (#STA-330, Cell Biolabs, San Diego, CA). The concentration range of MDA standards was 0 to 125 μM. The absorbance was measured at 540 nm. The concentration of MDA was calculated based on the standard curve created from the concentration and absorbance of the respective standard and described as nmol/mg of protein, as previously described (Zhao et al., 2014).

PC was measured following OxiSelect Protein Carbonyl ELISA Kit (#STA-310, Cell Biolabs). All samples were diluted using PBS to reach the protein concentration of 10 μg/mL. The working range of standards was 0 to 7.5 nmol/mg protein. The absorbance was measured at 540 nm. The concentration of PC was calculated based on the standard curve created from the concentration and absorbance of the respective standard and described as nmol/mg of protein, as previously described (Zhao et al., 2013).

### Apparently ileal digestibility

The frozen ileal digesta samples were dried by the freeze dryer (24D × 48, Virtis, Gardiner, NY). Dried digesta and feed samples were ground to fine powder form and stored in plastic containers for further analysis. Titanium dioxide concentration in the feed and digesta was measured as previously described (Chen et al., 2017; Moita et al., 2021b). The working range of the standards was 0 to 10 mg of titanium dioxide. Samples were weighed around 0.5 g onto a tarred weighing paper and then placed into 75 mL digestion tubes. One Kjeltab tablet (Fisher Scientific, Hampton, NH) and five pieces of selenized boiling granules were added to each digestion tube to prevent explosive vaporization. After adding 10 mL of concentrated H_2_SO_4_ (sulfuric acid), all digestion tubes were vortexed immediately. Then the tubes were heated for 2.5 h at 420 °C under a fume hood. When tubes got cool after 30 min at room temperature, 2 mL of 30% H_2_O_2_ (hydrogen peroxide) was added to each tube 4 times and were vortexed until a yellow to orange color appeared. Deionized water was added until the volumetric mark was reached and then the tubes were covered and gently mixed. Then, 200 µL from the tubes were pipetted to a 96-well plate, which was read immediately at 410 nm. Titanium dioxide values were calculated based on the standard curve created from the concentration and absorbance of the respective standards.

The feed and digesta samples were weighed at around 0.5 g to analyze the nitrogen content using TruSpec N Nitrogen Determinator (LECO CN-2000, LECO Corp., St. Joseph, MI) to later obtain the CP (6.25 × N). Furthermore, feed and digesta samples were weighed for determining DM (Method 934.01, AOAC, 2006), ADF (Method 973.18, AOAC, 2016), NDF (Van Soest et al., 1991), and EE (Method 2003.06, AOAC, 2006). The AID of DM, CP, EE, NDF, and ADF was calculated using the following equation as previously described (Chen et al., 2017):


$$\begin{array}{l} AID\left(\%\right)=100\times \left\{1\left[\left(TiO_{2}feed/TiO_{2}digesta\right)\times \left(N\;digesta/N\;feed\right)\right]\right\}, \end{array}$$

where TiO_2_ feed represents the titanium concentration in the feed, TiO_2_ digesta is the titanium concentration in the ileal digesta, N feed represents the nutrient concentration in the feed, and N digesta is the nutrient concentration in the ileal digesta.

### Intestinal morphology and crypt cell proliferation

After being fixed in 10% formalin, two sections of mid-jejunum were placed in cassettes that were reserved with 70% of ethanol solution. The samples were sent to the North Carolina State University Histology Laboratory (Raleigh, NC). Then, the samples were dehydrated, embedded in paraffin, cut cross-section to 5 µm thick, and mounted on polylysine-coated slides. Slides were stained using hematoxylin and eosin dyes for morphology measurements, and Ki-67 immunohistochemistry assay to detect Ki-67 positive cells to total cells in the crypt (%). Villus height, villus width, and crypt depth were measured using a microscope Olympus CX31 (Lumenera Corporation, Ottawa, Ontario, Canada) with a camera Infinity 2-2 digital CCD. Lengths of 10 well-oriented intact villi and their associated crypts were measured on each slide at a magnification of 40×. The villi length was measured from the top of the villi to the villi–crypt junction, the villi width was measured in the middle of the villi, and the crypt depth was measured from the villi–crypt junction to the bottom of the crypt. Then, the VH:CD was calculated. Images of 10 intact crypts from each slide, taken at a magnification of 100× were cropped and used for determining the enterocyte proliferation rate by analyzing the percentage of Ki-67 positive cells using the ImageJS software (Jang and Kim, 2019; Jang et al., 2020). The averages of the 10 measurements per pig were calculated and reported as 1 number per pig. The averages of the 10 measurements per pig were used 1 unit for statistical analysis. All analyses of the intestinal morphology were executed by the same person, as previously described (Shen et al., 2014).

### Statistical analysis

The data were analyzed based on a randomized complete block design by the SAS 9.4 software (SAS Inc., Cary, NC). Dietary treatments were considered fixed effects and the initial BW and sex blocks were considered random effects. Each treatment had 12 replicates (*n* = 12; and 3 BW blocks within sex). The experimental unit was the pig, individually housed and fed. The analyses of relative abundance and diversity of mucosa-associated microbiota in the jejunum, growth performance, nutrient digestibility, intestinal morphology, and immune and oxidative markers were performed using the MIXED procedure. The linear and quadratic effects of increasing levels of xylanase were tested by polynomial contrasts. Preplanned contrasts were made to evaluate the effects of the dietary inclusion of xylanase compared with no inclusion (0 vs. Xyl). When significant or tendency effects were found, the data were further analyzed using the NLMIXED procedure to determine the break point to obtain the optimal xylanase supplemental level, as previously described (Shen et al., 2012; Moita et al., 2021a). The predictor was set by multiplying the xylanase inclusion (XU per kg feed) with the ADFI (0.598 kg/d) to account for the feed consumption of the animals through the experimental period (XU per d). After the break point was found, it was converted back from XU per d to XU per kg feed by dividing with the ADFI (0.598 kg/d). For the broken-line model, the *P*-value of each parameter is indicated if the changes in the parameters are associated with the changes in the response. Statistical differences were considered significant with *P* < 0.05 and tendency with 0.05 ≤ *P* < 0.10.

## Results

### Growth performance

**Figure 1.**
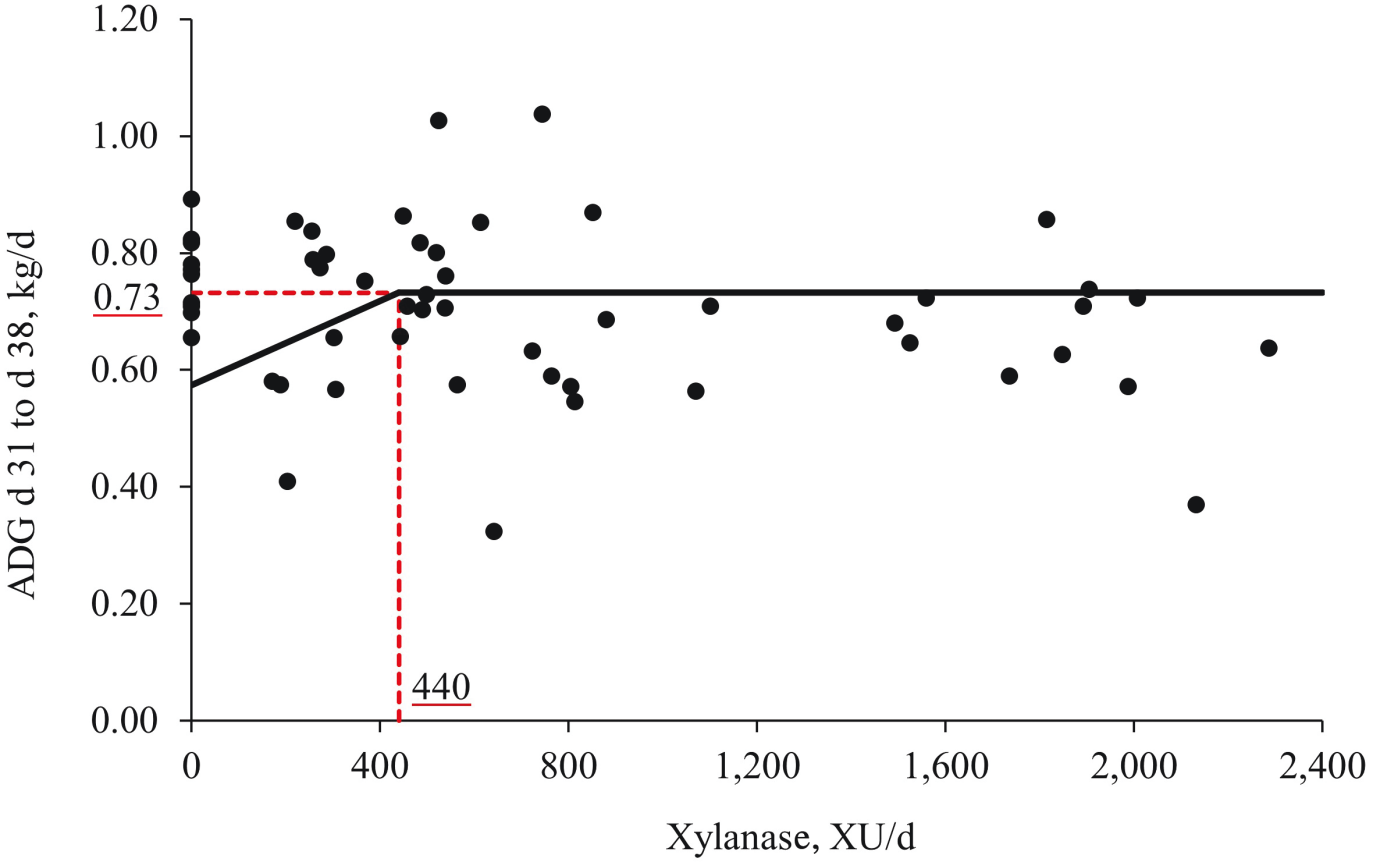
Changes in the ADG from days 31 to 38 with supplementation of xylanase using a broken-line analysis. The break point was 440 XU per d of xylanase supplementation when ADG from days 31 to 38 was 0.73 kg/d. The equation for ADG from days 31 to 38 was *Y* = 0.73 − 0.00036*x* zl; if xylanase supplementation is ≥break point, then z = 0; if xylanase supplementation is <break point, then zl = break point − xylanase supplementation. Values for xylanase activity were based on the analyzed values. *P*-value for the plateau was <0.0001, for the slope was 0.043, and for the breaking point was 0.012. The break point was converted from 440 XU per d to 736 XU per kg feed by dividing with the overall average feed intake (0.598 kg/d).

Increasing levels of xylanase in the diet of nursery pigs did not affect the BW and G:F during the experimental period (Table 3). Additionally, increasing levels of xylanase tended to increase (*P* = 0.057) the ADG from days 31 to 38. Increasing levels of xylanase tended to increase (*P* = 0.057) the ADFI from days 25 to 31 and increased (*P* < 0.05) the ADFI from days 31 to 38. Xylanase supplementation tended to reduce (*P* = 0.053) the BW of pigs at day 10, decreased (*P* < 0.05) the ADG and tended (*P* = 0.070) to decrease the ADFI from days 1 to 10 when compared with diet without xylanase supplementation (0 vs. Xyl). Conversely, xylanase supplementation increased (*P* < 0.05) the ADG and ADFI from days 31 to 38 and increased when compared with diet without xylanase supplementation (0 vs. Xyl). Additionally, xylanase supplementation increased (*P* < 0.05) the G:F from days 11 to 24 when compared with diet without xylanase supplementation (0 vs. Xyl). The broken-line analysis on the ADG from days 31 to d 38 in pigs fed diets with different xylanase supplemental levels indicated that the optimal xylanase level is 440 XU per d or 736 XU per kg feed (Figure 1).

**Table 3.** Growth performance of nursery pigs fed diets with increasing levels of xylanase

Item	Xylanase, XU per kg feed					SEM	*P*-value		
	0	220	440	880	1,760		Linear	Quad^1^	0 vs. Xyl^2^
BW, kg									
Days 0 to 1	6.9	6.9	6.8	6.9	6.9	0.6	0.923	0.929	0.842
Day 10	7.7	7.3	7.1	7.4	7.2	0.69	0.310	0.279	0.053
Day 24	12.5	12.5	11.9	12.4	12.4	1.05	0.929	0.536	0.704
Day 31	16.5	16.1	14.9	15.8	16.2	1.35	0.949	0.236	0.372
Day 38	20.7	20.8	19.9	20.7	21.4	1.58	0.566	0.464	0.944
ADG, g/d									
Days 1 to 10	78	39	26	45	35	26	0.281	0.247	0.040
Days 11 to 24	342	371	343	357	374	34	0.491	0.867	0.544
Days 25 to 31	579	591	575	595	639	40	0.231	0.667	0.622
Days 31 to 38	596	675	715	702	746	52	0.057	0.337	0.038
Overall	361	364	345	363	383	22	0.397	0.558	0.817
ADFI, g/d									
Days 1 to 10	152	106	105	111	119	32	0.589	0.172	0.070
Days 11 to 24	489	470	425	468	483	43	0.812	0.320	0.449
Days 25 to 31	943	1,007	1,019	1,031	1,102	79	0.057	0.770	0.120
Days 31 to 38	1,079	1,180	1,309	1,200	1,338	100	0.023	0.472	0.021
Overall	578	593	582	605	633	48	0.131	0.879	0.419
G:F									
Days 1 to 10	0.52	0.35	0.25	0.41	0.30	0.13	0.421	0.530	0.135
Days 11 to 24	0.69	0.78	0.80	0.76	0.78	0.04	0.369	0.288	0.047
Days 25 to 31	0.61	0.60	0.57	0.58	0.58	0.04	0.478	0.432	0.331
Days 31 to 38	0.55	0.57	0.55	0.59	0.56	0.04	0.944	0.527	0.713
Overall	0.61	0.62	0.60	0.60	0.61	0.03	0.815	0.717	0.866

Quadratic.

Xylanase (220 + 440 + 880 + 1,760 XU per kg feed).

### Digesta viscosity

**Figure 2.**
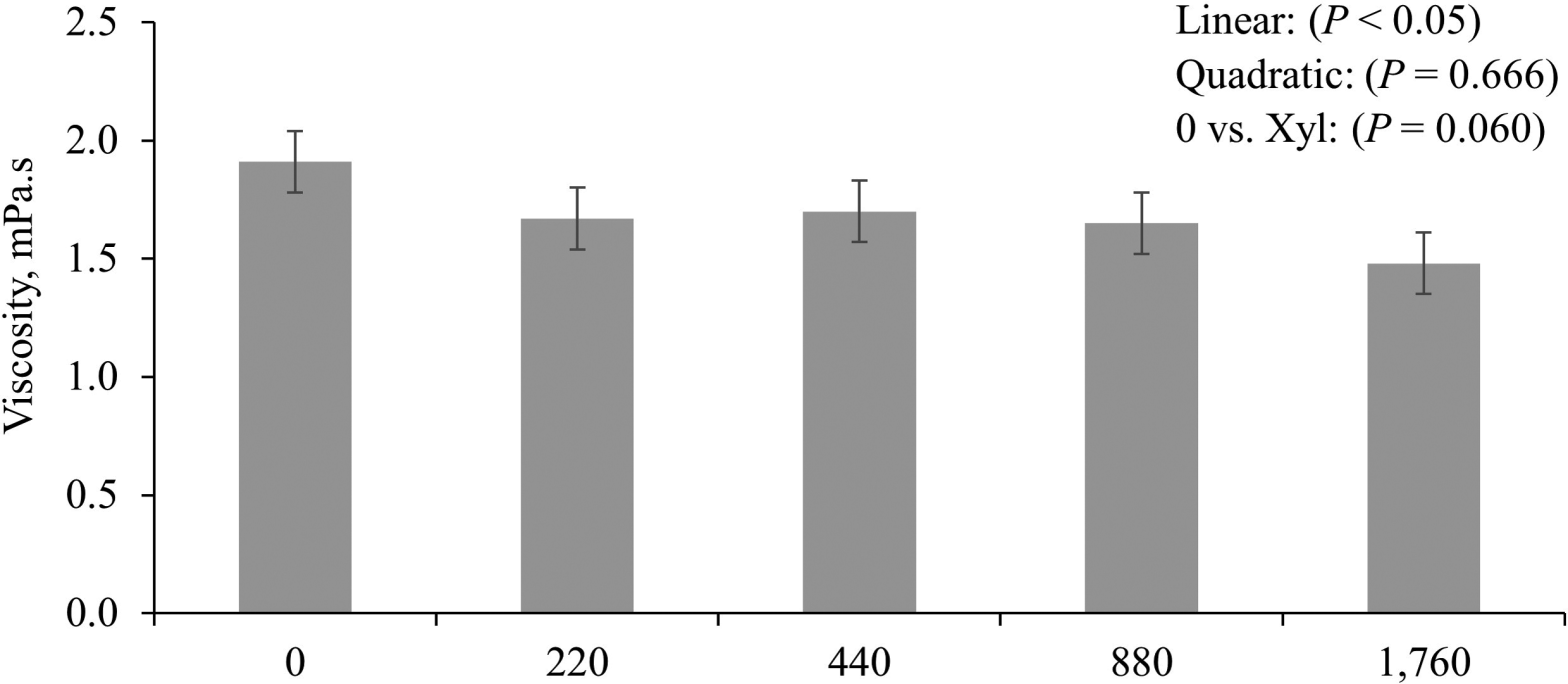
Changes in the viscosity of jejunal digesta of pigs fed diets with increasing levels of xylanase.

Increasing levels of xylanase in the diets of nursery pigs reduced (*P* < 0.05) the viscosity of jejunal digesta (Figure 2).

### Relative abundance and diversity of the mucosa-associated microbiota in jejunum

At the phylum level (Table 4), increasing levels of xylanase in the diet of nursery pigs did not affect the relative abundance of Proteobacteria, Firmicutes, Bacteriodetes, Actinobacteria, and Others (combined phyla with relative abundance lower than 1%) in jejunal mucosa of nursery pigs. At the family level (Table 5), increasing levels of xylanase affected (quadratic; *P* < 0.05) the relative abundance of *Moraxellaceae* (maximum: 2.51% at 825 XU per kg feed) and tended to increase (*P* = 0.096) the relative abundance of *Succinivibrionaceae*. Xylanase supplementation tended to decrease (*P* = 0.058) the relative abundance of *Halomonadaceae* and reduced (*P* < 0.05) the relative abundance of *Micrococcaceae* when compared with diet without xylanase supplementation (0 vs. Xyl).

**Table 4.** Relative abundance of jejunal mucosa-associated microbiota at the phylum level in nursery pigs fed diets with increasing levels of xylanase

Item	Xylanase, XU per kg feed					SEM	*P*-value		
	0	220	440	880	1,760		Linear	Quad^1^	0 vs. Xyl^2^
Proteobacteria	43.73	49.07	54.25	43.60	56.14	13.83	0.508	0.873	0.507
Firmicutes	36.03	38.69	24.02	27.79	19.21	8.53	0.125	0.757	0.376
Bacteriodetes	12.03	5.54	12.61	21.03	16.36	8.26	0.184	0.371	0.741
Actinobacteria	6.90	5.69	8.16	5.98	5.95	1.97	0.682	0.885	0.815
Others	1.66	1.00	0.96	1.59	2.34	0.64	0.117	0.314	0.743
F:B^3^	5.10	43.58	4.23	2.89	2.06	17.10	0.395	0.914	0.676

Quadratic.

Xylanase (220 + 440 + 880 + 1,760 XU per kg feed).

Firmicutes to Bacteroidetes ratio.

**Table 5.** Relative abundance of jejunal mucosa-associated microbiota at the family level in nursery pigs fed diets with increasing levels of xylanase

Item	Xylanase, XU per kg feed					SEM	*P*-value		
	0	220	440	880	1,760		Linear	Quad^1^	0 vs. Xyl^2^
*Clostridiaceae*	17.50	18.79	7.44	7.65	7.07	5.75	0.147	0.339	0.270
*Prevotellaceae*	10.18	4.39	10.20	17.51	14.24	7.80	0.223	0.506	0.802
*Helicobacteraceae*	9.24	17.91	8.59	13.53	10.87	11.14	0.942	0.862	0.738
*Comamonadaceae*	7.39	5.59	7.44	4.53	5.12	1.54	0.284	0.580	0.327
*Veillonellaceae*	4.44	1.66	3.53	3.42	1.93	1.59	0.321	0.896	0.159
*Lactobacillaceae*	4.24	3.21	4.22	1.08	1.44	1.97	0.239	0.653	0.435
*Enterobacteriaceae*	3.56	2.85	3.46	1.51	3.53	1.06	0.921	0.221	0.550
*Pseudomonadaceae*	3.09	3.62	8.23	3.41	8.12	1.83	0.109	0.947	0.191
*Oxalobacteraceae*	3.03	5.72	9.52	1.53	10.47	4.79	0.205	0.514	0.254
*Microbacteriaceae*	2.68	1.81	2.60	1.65	1.73	0.50	0.224	0.580	0.205
*Halomonadaceae*	2.58	0.02	0.08	0.04	0.03	1.14	0.298	0.244	0.058
*Erysipelotrichaceae*	2.34	5.69	0.61	7.13	1.18	3.57	0.780	0.395	0.728
*Peptostreptococcaceae*	2.29	1.68	0.48	1.49	2.01	0.88	0.886	0.273	0.385
*Burkholderiaceae*	1.79	1.47	1.99	1.03	1.89	0.34	0.142	0.622	0.348
*Micrococcaceae*	1.17	0.30	0.55	0.28	0.46	0.29	0.266	0.117	0.025
*Sphingomonadaceae*	1.57	1.67	2.57	1.40	2.12	0.46	0.612	0.989	0.451
*Lachnospiraceae*	1.41	0.98	1.73	1.63	1.44	0.74	0.754	0.565	0.948
*Methylobacteriaceae*	1.34	1.61	1.73	0.75	1.54	0.48	0.862	0.488	0.910
*Moraxellaceae*	1.22	1.52	2.45	2.37	0.62	1.01	0.373	0.038	0.471
*Campylobacteraceae*	1.19	0.80	0.75	2.47	0.44	0.77	0.774	0.156	0.923
*Propionibacteriaceae*	1.19	1.68	2.31	2.45	1.48	0.85	0.887	0.111	0.283
*Eubacteriaceae*	1.10	1.46	1.12	1.14	0.56	0.67	0.307	0.646	0.964
*Succinivibrionaceae*	1.04	0.73	0.81	3.63	2.44	1.00	0.096	0.277	0.424
*Xanthomonadaceae*	0.94	2.29	3.74	0.60	3.85	1.48	0.235	0.656	0.197
*Caulobacteraceae*	0.70	0.78	0.40	0.34	0.91	0.49	0.666	0.175	0.782
*Ruminococcaceae*	0.68	1.11	1.04	1.10	1.33	0.62	0.401	0.808	0.374
*Porphyromonadaceae*	0.64	0.34	0.97	1.12	0.65	0.38	0.630	0.113	0.667
*Pasteurellaceae*	0.32	0.45	0.29	4.21	0.67	1.83	0.645	0.208	0.603
*Streptococcaceae*	0.29	0.84	0.99	0.71	0.28	0.35	0.460	0.121	0.238
*Bacillaceae*	0.12	1.82	0.35	0.22	0.21	0.73	0.502	0.943	0.507
Others	10.70	7.18	9.78	10.05	12.06	3.56	0.414	0.669	0.758

Quadratic.

Xylanase (220 + 440 + 880 + 1,760 XU per kg feed).

At the genus level (Table 6), increasing levels of xylanase tended to decrease the relative abundance of *Cupriavidus* (*P* = 0.073) and *Megasphaera* (*P* = 0.063). Increasing levels of xylanase tended to increase the relative abundance of *Succinivibrio* (*P* = 0.076) and *Pseudomonas* (*P* = 0.060). Moreover, increasing levels of xylanase affected (quadratic; *P* < 0.05) the relative abundance of *Acinetobacter* (maximum: 2.01% at 867 XU per kg feed), whereas xylanase supplementation decreased (*P* < 0.05) the relative abundance of *Cupriavidus*, *Megasphaera*, and *Arthrobacter* when compared with diet without xylanase supplementation (0 vs. Xyl).

**Table 6.** Relative abundance of jejunal mucosa-associated microbiota at the genus level in nursery pigs fed diets with increasing levels of xylanase

Item	Xylanase, XU per kg feed					SEM	*P*-value		
	0	220	440	880	1,760		Linear	Quad^1^	0 vs. Xyl^2^
*Clostridium*	17.40	18.37	6.70	7.88	8.78	6.70	0.311	0.355	0.362
*Helicobacter*	13.34	19.59	10.47	14.09	12.59	11.99	0.845	0.969	0.940
*Prevotella*	10.13	4.09	10.17	16.84	17.36	8.65	0.234	0.620	0.827
*Pelomonas*	9.04	6.74	8.76	5.85	5.71	2.00	0.224	0.694	0.294
*Microbacterium*	3.53	3.39	3.38	2.20	2.22	0.75	0.226	0.621	0.216
*Turicibacter*	2.59	5.61	0.22	7.47	0.85	4.04	0.747	0.421	0.819
*Massilia*	2.14	3.99	7.76	0.97	7.84	3.79	0.232	0.595	0.258
*Campylobacter*	1.45	1.04	1.01	2.54	0.62	0.85	0.697	0.219	0.853
*Selenomonas*	1.40	0.11	0.71	1.75	0.46	0.88	0.805	0.534	0.400
*Cupriavidus*	1.33	0.66	1.20	0.63	0.70	0.20	0.073	0.286	0.027
*Acinetobacter*	1.28	2.05	2.88	3.41	0.78	1.51	0.575	0.042	0.371
*Herbaspirillum*	1.28	1.55	1.71	0.51	2.51	0.81	0.251	0.155	0.671
*Sphingomonas*	1.27	1.08	1.50	1.01	1.06	0.25	0.464	0.991	0.697
*Megasphaera*	1.26	0.53	0.54	0.28	0.23	0.31	0.063	0.184	0.023
*Arthrobacter*	1.14	0.41	0.41	0.34	0.45	0.28	0.259	0.117	0.027
*Mitsuokella*	1.10	0.65	1.48	0.71	0.80	0.54	0.665	0.990	0.731
*Succinivibrio*	1.10	0.71	0.82	4.04	2.71	1.04	0.076	0.276	0.395
*Ralstonia*	0.53	0.79	0.68	0.42	0.55	0.19	0.528	0.810	0.679
*Roseburia*	0.47	0.35	0.88	0.82	0.66	0.56	0.629	0.442	0.621
*Actinobacillus*	0.42	0.48	0.27	3.40	0.65	1.48	0.662	0.238	0.644
*Bifidobacterium*	0.36	0.38	1.49	0.90	0.22	0.53	0.532	0.639	0.769
*Streptococcus*	0.35	1.24	1.20	0.90	0.36	0.51	0.474	0.185	0.262
*Telluria*	0.27	0.53	0.89	0.12	1.09	0.52	0.100	0.377	0.197
*Pseudomonas*	4.89	5.80	12.44	5.03	13.29	3.09	0.060	0.757	0.160
*Lactobacillus*	4.84	3.98	4.86	1.21	1.66	2.42	0.256	0.671	0.487
*Methylobacterium*	1.77	2.23	2.08	1.00	1.94	0.67	0.787	0.479	0.958
*Propionibacterium*	1.66	2.46	2.98	3.50	2.04	1.24	0.889	0.124	0.300
Others	13.63	12.09	12.50	13.08	14.45	5.37	0.707	0.755	0.868

Quadratic.

Xylanase (220 + 440 + 880 + 1,760 XU per kg feed).

At the species level (Table 7), increasing levels of xylanase decreased (*P* < 0.05) the relative abundance of *Cupriavidus necator*, increased (*P* < 0.05) the relative abundance of *Massilia indica* and tended to increase (*P* = 0.055) the relative abundance of *Telluria mixta*. Increasing levels of xylanase affected (quadratic; *P* < 0.05) the relative abundance of *C. necator* (minimum: 0.29% at 1,250 XU per kg feed), *Campylobacter coli* (maximum: 1.54% at 925 XU per kg feed) and tended to affect the relative abundance of *Succinivibrio dextrinosolvens* (quadratic; *P* = 0.086; maximum: 1.98% at 900 XU per kg feed) and *Acinetobacter johnsonii* (quadratic; *P* = 0.096; maximum: 1.35% at 950 XU per kg feed). Furthermore, xylanase supplementation tended to reduce the relative abundance of *Microbacterium ginsengisoli* (*P* = 0.098) and *Campylobacter upsaliensis* (*P* = 0.051) and reduced (*P* < 0.05) the relative abundance of *C. necator* when compared with diet without xylanase supplementation (0 vs. Xyl). The alpha diversity of jejunal mucosa-associated microbiota estimated with Chao1 richness, Shannon diversity, and Simpson diversity at family and genus levels was not affected by dietary xylanase supplementation (Table 8).

**Table 7.** Relative abundance of jejunal mucosa-associated microbiota at the species level in nursery pigs fed diets with increasing levels of xylanase

Item	Xylanase, XU per kg feed					SEM	*P*-value		
	0	220	440	880	1,760		Linear	Quad^1^	0 vs. Xyl^2^
*Prevotella copri*	11.94	5.40	11.54	19.66	17.42	10.10	0.240	0.652	0.827
*Pelomonas puraquae*	9.82	7.14	8.73	4.87	5.70	2.17	0.178	0.408	0.199
*Clostridium butyricum*	8.56	8.05	1.46	1.82	3.76	3.63	0.332	0.196	0.247
*Helicobacter mastomyrinus*	5.40	9.89	7.68	3.85	7.85	5.85	0.985	0.765	0.712
*Helicobacter rappini*	5.35	12.07	7.64	7.27	7.48	7.77	0.930	0.874	0.616
*Microbacterium ginsengisoli*	4.69	2.71	3.85	2.35	2.84	0.91	0.252	0.289	0.098
*Pelomonas aquatica*	3.61	2.31	3.13	1.93	2.30	0.81	0.330	0.410	0.202
*Propionibacterium acnes*	3.60	4.67	5.51	6.13	4.17	2.19	0.908	0.271	0.456
*Turicibacter sanguinis*	2.46	8.35	0.18	7.26	0.65	6.11	0.630	0.521	0.743
*Campylobacter upsaliensis*	1.88	0.27	0.42	0.26	0.17	0.69	0.226	0.266	0.051
*Clostridium perfringens*	1.66	0.85	0.46	0.44	0.31	0.71	0.268	0.398	0.168
*Prevotellasp.*	1.50	0.58	1.08	1.20	1.49	0.59	0.515	0.517	0.420
*Prevotella stercorea*	1.50	0.58	1.08	1.20	1.49	0.59	0.515	0.517	0.420
*Cupriavidus necator*	1.34	0.60	0.96	0.35	0.59	0.18	0.019	0.023	0.001
*Arthrobacter russicus*	1.31	0.82	0.65	0.56	0.81	0.48	0.594	0.310	0.265
*Dialister succinatiphilus*	1.04	0.20	1.17	0.47	0.61	0.54	0.742	0.823	0.494
*Succinivibrio dextrinosolvens*	0.92	0.64	0.83	2.94	1.20	0.73	0.333	0.086	0.515
*Massiliasp.*	0.90	1.15	1.99	0.89	2.67	1.25	0.213	0.274	0.526
*Mitsuokella multacida*	0.85	0.46	0.88	0.30	0.57	0.44	0.668	0.618	0.551
*Lactobacillus mucosae*	0.85	1.58	1.14	0.26	0.49	0.72	0.373	0.820	0.981
*Mitsuokella jalaludinii*	0.64	0.42	1.07	0.33	0.59	0.34	0.801	0.934	0.926
*Roseburia faecis*	0.59	0.42	1.10	1.00	0.83	0.68	0.583	0.459	0.612
*Helicobactersp.*	0.58	1.46	0.10	2.98	0.49	1.57	0.996	0.204	0.499
*Pseudomonas caricapapayae*	0.57	0.56	0.67	0.52	1.12	0.30	0.185	0.479	0.665
*Telluria mixta*	0.47	0.83	1.70	0.19	2.20	1.02	0.055	0.346	0.189
*Faecalibacterium prausnitzii*	0.45	0.81	0.58	0.28	0.49	0.27	0.630	0.719	0.771
*Acinetobacter johnsonii*	0.45	0.80	1.08	1.22	0.29	0.62	0.636	0.096	0.424
*Clostridiumsp.*	0.50	2.09	3.24	0.53	0.76	1.30	0.520	0.617	0.470
*Campylobacter coli*	0.30	0.31	0.24	2.26	0.75	0.77	0.816	0.034	0.537
*Massilia varians*	0.30	0.22	1.31	0.95	1.17	0.58	0.243	0.740	0.417
*Pseudomonas hibiscicola*	0.26	0.60	1.40	0.14	1.72	0.80	0.113	0.567	0.233
*Lactobacillus delbruecki*	0.19	1.76	0.30	0.22	0.38	0.77	0.621	0.792	0.625
*Massilia alkalitolerans*	0.18	0.28	1.33	0.42	1.04	0.54	0.269	0.891	0.265
*Campylobacter hyointestinalis*	0.08	0.43	0.40	1.56	0.13	0.71	0.857	0.118	0.451
*Massilia indica*	0.05	0.75	0.80	0.24	1.74	0.70	0.047	0.506	0.139
Others	26.75	20.23	25.91	24.66	25.83	6.18	0.841	0.854	0.683

Quadratic.

Xylanase (220 + 440 + 880 + 1,760 XU per kg feed).

**Table 8.** Alpha diversity of jejunal mucosa-associated microbiota at the family and genus level in nursery pigs fed diets with increasing levels of xylanase

Item	Xylanase, XU per kg feed					SEM	*P*-value		
	0	220	440	880	1,760		Linear	Quad^1^	0 vs. Xyl^2^
Family level									
Chao1	69.33	70.67	71.67	81.48	76.17	9.89	0.346	0.395	0.463
Shannon	4.08	3.52	4.17	3.70	3.89	0.55	0.905	0.755	0.546
Simpson	0.87	0.77	0.88	0.78	0.84	0.07	0.943	0.496	0.474
Genus level									
Chao1	78.17	71.17	81.83	85.19	80.50	11.05	0.587	0.546	0.873
Shannon	3.99	3.37	4.04	3.54	3.68	0.56	0.752	0.751	0.466
Simpson	0.83	0.74	0.86	0.75	0.81	0.08	0.907	0.652	0.564

Quadratic.

Xylanase (220 + 440 + 880 + 1,760 XU per kg feed).

### Inflammatory and oxidative stress parameters

**Figure 3.**
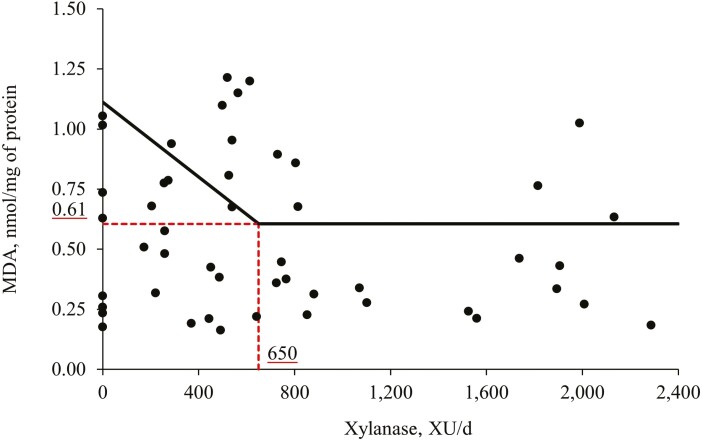
Changes in the MDA concentration with supplementation of xylanase using a broken-line analysis. The break point was 650 XU per d of xylanase supplementation when MDA concentration was 0.61 µmol/g of protein. The equation for MDA concentration was *Y* = 0.61 + 0.00078*x* zl; if xylanase supplementation is ≥break point, then z = 0; if xylanase supplementation is <break point, then zl = break point − xylanase supplementation. Values for xylanase activity were based on the analyzed values. *P*-value for the plateau was <0.0001, for the slope was 0.020, and for the breaking point was 0.005. The break point was converted from 650 XU per d to 1,087 XU per kg feed by dividing with the overall average feed intake (0.598 kg/d).

Increasing levels of xylanase did not affect the immune status parameters in jejunal mucosa of nursery pigs (Table 9). However, increasing levels of xylanase in the diet of nursery pigs tended to affect (quadratic; *P* = 0.059) the concentration of PC, whereas the xylanase supplementation reduced (*P* < 0.05) the concentration of PC in the jejunal mucosa when compared with diet without xylanase supplementation (0 vs. Xyl). Additionally, increasing levels of xylanase reduced (*P* < 0.05) the concentration of MDA in jejunal mucosa of nursery pigs. The broken-line analysis on the MDA concentration in pigs fed diets with increasing levels of xylanase indicated that the optimal xylanase level is 650 XU per d or 1,087 XU per kg feed (Figure 3).

**Table 9.** Concentrations of pro-inflammatory cytokines and oxidative stress products in the jejunal mucosa of nursery pigs fed diets with increasing levels of xylanase

Item^1^	Xylanase, XU per kg feed					SEM	*P*-value		
	0	220	440	880	1,760		Linear	Quad^2^	0 vs. Xyl^3^
IL-8, ng/mg of protein	0.70	0.61	0.54	0.64	0.72	0.11	0.404	0.138	0.314
TNF-α, pg/mg of protein	0.55	0.59	0.56	0.49	0.51	0.11	0.503	0.836	0.867
Protein carbonyl, nmol/mg of protein	2.29	1.86	1.92	1.82	1.95	0.20	0.329	0.059	0.017
MDA, nmol/mg of protein	0.99	0.97	0.87	0.58	0.60	0.30	0.045	0.390	0.223

IL-8, interleukin-8; TNFa, tumor necrosis factor-alpha; MDA, malondialdehyde.

Quadratic.

Xylanase (220 + 440 + 880 + 1,760 XU per kg feed).

### Apparent ileal digestibility

**Figure 4.**
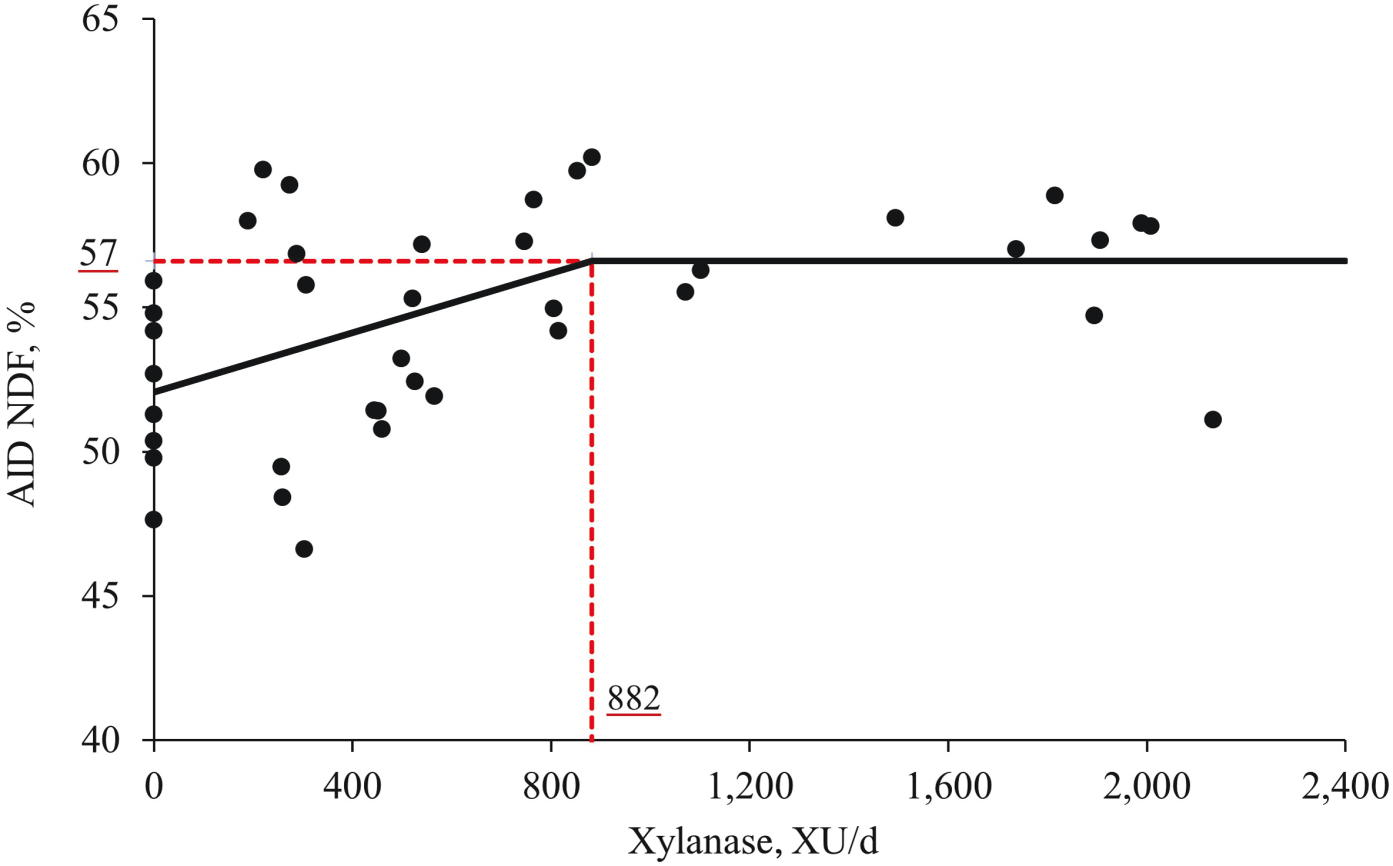
Changes in the AID of NDF with supplementation of xylanase using a broken-line analysis. The break point was 882 XU per d of xylanase supplementation when the AID of NDF was 57%. The equation for AID of NDF was *Y* = 57 − 0.00515*x* zl; if xylanase supplementation is ≥break point, then z = 0; if xylanase supplementation is <break point, then zl = break point − xylanase supplementation. Values for xylanase activity were based on the analyzed values. *P*-value for the overall model was <0.001, for the plateau was <0.0001, for the slope was 0.001, and for the breaking point was <0.001. The break point was converted from 882 XU per d to 1,475 XU per kg feed by dividing with the overall average feed intake (0.598 kg/d).

Increasing levels of xylanase tended to increase (*P* = 0.058) the AID of CP and increased (*P* < 0.05) the AID of EE and NDF (Table 10). The xylanase supplementation did not affect the AID of DM and ADF. The broken-line analysis on the AID of NDF in pigs fed diets with increasing levels of xylanase indicated that the optimal xylanase level is 882 XU per d or 1,475 XU per kg feed (Figure 4).

**Table 10.** Apparent ileal digestibility of dry matter, crude protein, acid detergent fiber, neutral detergent fiber, and ether extract in feeds fed to nursery pigs with increasing levels of xylanase

Item^1^	Xylanase, XU per kg feed					SEM	*P*-value		
	0	220	440	880	1,760		Linear	Quad^2^	0 vs. Xyl^3^
AID, %									
DM	59.95	58.92	63.38	62.63	63.47	1.78	0.102	0.404	0.287
CP	71.41	74.34	72.61	75.59	76.71	2.15	0.058	0.644	0.115
NDF	52.95	54.27	52.68	56.12	56.17	1.46	0.036	0.611	0.167
ADF	35.29	35.43	35.55	36.04	37.37	1.26	0.125	0.805	0.486
EE	83.90	84.01	84.72	87.57	89.50	3.20	0.024	0.845	0.264

AID, apparent ileal digestibility; DM, dry matter; CP, crude protein; NDF, neutral detergent fiber; ADF, acid detergent fiber; EE, ether extract.

Quadratic.

Xylanase (220 + 440 + 880 + 1,760 XU per kg feed).

### Intestinal morphology and crypt cell morphology

Xylanase supplementation tended to increase (*P* = 0.073) the villus height when compared with diet without xylanase supplementation (0 vs. Xyl; Table 11). Increasing levels of xylanase tended to affect (quadratic; *P* = 0.060; maximum: 109 μm at 922 XU per kg feed) the villus width in the jejunum of pigs. However, xylanase supplementation did not affect crypt depth and the ratio of Ki-67 positive cells.

**Table 11.** Morphology and crypt cell proliferation in the jejunum of nursery pigs fed diets with increasing levels of xylanase

Item	Xylanase, XU per kg feed					SEM	*P*-value		
	0	220	440	880	1,760		Linear	Quad^1^	0 vs. Xyl^2^
Villus height, μm	314	350	357	355	363	20	0.169	0.310	0.073
Villus width, μm	96	97	108	107	104	5	0.197	0.060	0.114
Crypt depth, μm	110	108	109	114	121	7	0.107	0.736	0.659
VH:CD ratio^3^	2.95	3.27	3.29	3.17	3.03	0.21	0.774	0.324	0.323
Ki-67 positive, %^4^	37.2	37.3	39.5	37.1	33.5	2.5	0.123	0.289	0.862

Quadratic.

Xylanase (220 + 440 + 880 + 1,760 XU per kg feed).

Villus height to crypt depth ratio.

Ratio of Ki-67 positive cells to total cells in the crypt.

## Discussion

In this study, the supplementation of increasing dietary levels of xylanase reduced the jejunal digesta viscosity of nursery pigs. The effectiveness of xylanase reducing the digesta viscosity has been reported by different authors and illustrates the reduction of the negative impacts of the xylan associated with digesta viscosity, microbiota composition, immune response, oxidative stress status, nutrient digestibility, and intestinal morphology (Duarte et al., 2019; Chen et al., 2020; Petry et al., 2021). The viscosity of digesta can be affected due to the presence of considerable amounts of soluble NSP in most of the plant-based feedstuffs, such as xylan, arabinoxylan, xyloglucans, β-glucans, and others (Tiwari et al., 2018; Baker et al., 2021). These structures cannot be degraded by endogenous enzymes secreted by pigs and may pass through the gastrointestinal tract completely undigested (Qi et al., 2018; Baker et al., 2021). Cereal grains typically used in pig diets can contain high levels of soluble NSP (Choct, 2015; Baker et al., 2021). Corn and DDGS that are usually present in typical nursery diets have been reported to present high levels of NSP comprised in their composition (Choct, 2015). The DDGS may present a higher NSP content due to the ethanol manufacturing process, where there is an increase in the solubility of xylose and arabinose leading to negative effects associated with digesta viscosity and nutrient digestibility (Pedersen et al., 2014). The negative effects of the soluble NSP, especially xylans, on digesta viscosity can be related to the chemical structure and molecular weight of the specific polysaccharide rather than linkage type and sugar composition (Choct, 2015; Baker et al., 2021). Xylans can increase the digesta viscosity leading to a reduction on the enzymatic activity resulting in a decreased digestibility of dietary components such as GE, DM, CP, amino acids, and minerals (Gutierrez et al., 2014; Jaworski et al., 2015; Baker et al., 2021). In addition, they possess greater swelling and water-holding capacity and solubility that will result in increased viscosity of the digesta and can also increase the passage rate and digesta bulk (Tervilä-Wilo et al., 1996; O’Neill et al., 2012; Duarte et al., 2019). An increase in the digesta bulk may cause a distention on the wall of the digestive tract that can lead to an increase in the secretion of cholecystokinin, a hormone that plays a role in satiety and stimulating pancreatic secretions (McDonald et al., 2010; Duarte et al., 2019). This could increase the endogenous losses and lead to negative effects on the intestinal health and growth performance (Agyekum and Nyachoti, 2017).

The increased digesta viscosity can also exert an effect on the modulation of the mucosa-associated microbiota in the jejunum by potentially increasing the presence of pathogens, such as *Escherichia coli* and *Clostridium perfringens* (Annett et al., 2002; Hopwood and Hampson, 2003). The hydrolysis of xylan by xylanase may play an important role in the modulation of the gastrointestinal physiology and microbiota by providing forms of more fermentable NSP-released compounds, such as xylooligosaccharides (Baker et al., 2021; Duarte and Kim, 2021; Petry et al., 2021). The microbiota can be affected by dietary components at different taxonomic levels (Duarte and Kim, 2021). In this study, xylanase supplementation affected the microbiota at lower levels (genus and species) to a greater extent than the family level and had no effect at the phylum level, which may be partially explained by the products released by xylanase that selectively modulate the microbiota (Baker et al., 2021; Petry et al., 2021). In this study, the supplementation of xylanase reduced the relative abundance of *C. necator* and tended to increase the abundance of *Succinivibrio* and *Pseudomonas*. It was reported in human studies that a low abundance of *Succinivibrio* is correlated with gastrointestinal disorders and loss of intestinal integrity in the colon (Hespell, 1992). Moreover, *Succinivibrio* has been reported to degrade cellulose and hemicellulose (Pu et al., 2020). Although *Pseudomonas* belongs to Proteobacteria phylum, its ability to adapt to degrade fiber has been previously reported (Guo et al., 2018). The results reported in this study may indicate a change in the jejunal environment and substrates toward a healthier microbiome as a consequence of the positive effects observed in the nutrients digestibility and reduced viscosity. Therefore, the release of xylooligosaccharides and the improved nutrient digestibility by xylanase supplementation may benefit the growth of fiber-degrading bacteria, and competitively reducing the growth of protein-degrading bacteria (Zhang et al., 2018; Duarte and Kim, 2021; Duarte et al., 2021; Petry et al., 2021).

When enzyme activity is discussed, it is important to remember that enzyme activity is correlated and determined by the presence of the specific substrates that this enzyme will target under optimal conditions such as temperature and pH. In this study, the inclusion of feedstuffs containing soluble NSP, such as corn and DDGS, was increased throughout the nutritional phases, which may result in a higher efficacy of xylanase, as the animals started to consume more soluble NSP present in feedstuffs (Petry et al., 2020). The results of the present study showed that xylanase supplementation moderately improved the growth performance by increasing the ADFI and tending to increase the ADG during the last week of the study. This may indicate a high efficacy of xylanase during this period due to the presence of higher levels of soluble NSP and an adaption period longer than 14 days of xylanase feeding (Petry et al., 2020). In addition, it was found by broken-line analysis that the optimal supplemental level of xylanase for maximal ADG from days 31 to 38 was 440 XU per d or 736 XU per kg feed based on corn, soybean meal, and corn DDGS. The supplementation of xylanase will increase the hydrolysis of xylans and arabinoxylans (Duarte et al., 2019; Chen et al., 2020; Baker et al., 2021) that could result in the release of xylooligosaccharides. These compounds can exert positive effects by acting as prebiotics and leading to improvements in growth performance and nutrient digestibility (McDonald et al., 2001; Baker et al., 2021).

According to Duarte et al. (2021), the reduction of digesta viscosity and the release of entrapped nutrients may increase the interaction of endogenous enzymes and their substrates, favoring the digestion and absorption of nutrients in the intestinal tract. In this study, an increase in the AID of CP was observed, which indicates that it can be related with the decrease on the digesta viscosity leading to a reduced endogenous protein loss and a greater capacity of NSP-degrading enzymes to release nutrients from the cells through the gastrointestinal tract. Additionally, it was reported that NSP enzymes, such as β-glucanase and xylanase, can increase pepsin activity in the gastric mucosa and γ-glutamyl transpeptidase and disaccharidases in jejunal and ileal mucosa of nursery pigs fed a barley-based diets (Fan et al., 2009). The microbiota population in the small intestine has the ability to ferment proteins, although to a lesser extent (Dai et al., 2010; Davila et al., 2013; Duarte and Kim, 2021). The increased protein digestibility may have reduced the substrate for protein fermenting bacteria in the small intestine. These findings agree with the results of the present study, where it observed a tendency to increase on the relative abundance of *Succinivibrio* and a decrease on the relative abundance of *C. necator* in the jejunal mucosa, which may have exerted a positive effect on protein utilization.

The enhancement of the intestinal environment may also positively impact the nutrient digestibility leading to potential benefits associated with growth performance (Duarte et al., 2019, 2021; Chen et al., 2020. In this study, it was observed that xylanase supplementation increased the AID of NDF, which is in agreement with Passos et al. (2015) and Chen et al. (2020), where it was also observed a positive effect of xylanase on the AID of NDF. A possible mechanism may be through the modulation of the mucosa-associated microbiota. The supplementation of xylanase can generate compounds such as oligosaccharides, xylooligosaccharides, and arabinoxylooligosaccharides (Baker et al., 2021). These compounds can serve as fermentable substrates for specific bacterial species to help in their proliferation that in turn, will start to secrete their own fiber-degrading enzymes (Duarte et al., 2021) resulting in increased AID of NDF.

The increase in the relative abundance of *Succinivibrio*, which can be classified as a semi-cellulose bacterium, observed in the present study may provide positive effects by improving the fiber degradation which may reflect on an increase in the AID of NDF (Hespell, 1992). In addition, it was found by broken-line analysis that the optimal supplemental level of xylanase for improving AID of NDF was 882 XU per d or 1,475 XU per kg feed based on corn, soybean meal, and corn DDGS. Furthermore, an increase in the AID of EE was observed in this study. It is important to remember that pigs improve their ability to emulsify, digest, and absorb lipids and fatty acids as they grow (Diebold et al., 2004). In this regard, the viscosity may also have an impact on AID of EE. When the viscosity is high, there will be less interaction between the hydrophobic lipids and their emulsifiers, such as the bile salts (Costa et al., 2019). Since a reduction in the digesta viscosity was observed in the present study, this may partially explain the improvements observed with the AID of EE. Additionally, fat sources can play a role on the modulation of the microbiota (Yang et al., 2020). Some microorganisms are considered lipolytic bacteria, which means that they use hydrolysates such as glycerol and fatty acids to produce energy for epithelial cells (de Wit et al., 2012). Thereby, a positive modulation of the microbiota population and an increase in the relative abundance of lipolytic bacteria may also explain the benefits observed in the AID of EE.

Even though a potential improvement in growth performance and a reduced digesta viscosity of nursery pigs supplemented with xylanase were observed in the present study, no differences were observed on the inflammatory parameters from the jejunal mucosa. The results of the present study are in accordance with Duarte et al. (2019), where the authors also did not report any differences in the inflammatory parameters in the jejunal mucosa of the pigs. On the other hand, it was observed in the present study that xylanase supplementation affected the oxidative stress by reducing the concentration of MDA and PC on the jejunal mucosa. The MDA and PC are products of lipid peroxidation and protein oxidation, respectively. The results of the present study are in agreement with Duarte et al. (2019) and Petry et al. (2020), whereas Tiwari et al. (2018) did not find any impact associated with the oxidative stress.

The mechanism that xylanase modulates the oxidative stress status and improves antioxidant capacity remains unclear. According to Petry et al. (2020), a potential mechanism may be due to an increase in the bioavailability of phenolic compounds derived from the arabinoxylan structure. According to the same authors, the corn arabinoxylan structure may comprise different phenolic compounds such as caffeic acid, sinapic acid, and ferulic acid. Phenolic compounds, such as ferulic acid, are considered potent antioxidants (Ogiwara et al., 2002; Wang et al., 2020) and have antimicrobial properties (Borges et al., 2013) that can impact the microbiota population by reducing the relative abundance of enterotoxigenic *E. coli* K88 and F18^+^ in pig feces (Arzola-Alvarez et al., 2020). Ferulic acid was shown to have correlations to oxidative stress status in pigs (Petry et al., 2020; Wang et al., 2020). It is possible that xylanase supplementation increased the bioavailability of phenolic compounds, such as ferulic acid, by increasing the fragmentation of the arabinoxylan structure. Further research should be done in order to elucidate the mechanism and the effects of phenolic compounds associated with the oxidative stress of pigs. In addition, it was found by broken-line analysis that the optimal supplemental level of xylanase for reducing MDA concentration on the jejunal mucosa was 650 XU per d or 1,087 XU per kg feed based on corn, soybean meal, and corn DDGS.

According to Duarte et al. (2019), compounds generated from oxidative stress can negatively affect the cell integrity and intestinal morphology. In this study, an increase in the villus height and width was observed, which can be an indicator of improved intestinal health and enhanced nutrient absorption and utilization capacity. Therefore, the positive effects on the oxidative stress observed in this study may be partially explained by the improved intestinal morphology.

Other studies were recently published (Zhang et al., 2018; Lu et al., 2021; Petry et al., 2021) evaluating the supplemental effects of xylanase and the correlation with the intestinal microbiota, whereas their impacts associated with the modulation of the intestinal microbiota and their relationship with intestinal health and growth performance have not been well elucidated. In this study, the supplementation of xylanase showed potential benefits for intestinal health by reducing the digesta viscosity and also by positively modulating the mucosa-associated microbiota in the jejunum, which may have reflected in improvements related to intestinal health, nutrient digestibility, intestinal morphology, and growth performance of nursery pigs.

In conclusion, xylanase supplementation showed benefits on intestinal health by reducing digesta viscosity, the relative abundance of potentially harmful bacteria, and the oxidative stress in the jejunal mucosa, collectively enhancing intestinal morphology and the AID of nutrients. Xylanase supplementation at a range of 750 to 1,500 XU per kg feed based on corn, soybean meal, and corn DDGS provided benefits associated with reduced oxidative stress by reducing the MDA concentration on the jejunal mucosa, increased nutrient digestibility by improving the AID of CP, NDF, and EE, resulting in potential improvement on growth performance of nursery pigs by increasing the ADFI and moderately improving the ADG throughout the last week feeding.

## Abbreviations

ADF: acid detergent fiber
ADFI: average daily feed intake
ADG: average daily gain
AID: apparent ileal digestibility
AX: arabinoxylan
BW: body weight
CP: crude protein
DDGS: distillers’ dried grains with soluble
DM: dry matter
EE: ether extract
ELISA: enzyme-linked immunosorbent assay
G:F: gain to feed ratio
NDF: neutral detergent fiber
NSP: nonstarch polysaccharides
OTU: operational taxonomic unit
PBS: phosphate-buffered saline solution
PC: protein carbonyl
RA: relative abundance
TAXI: *Triticum aestivum* xylanase inhibitor
TiO_2_: titanium dioxide
XIP: xylanase inhibitor protein
